# Nutritional strategies in an elite wheelchair marathoner at 3900 m altitude: a case report

**DOI:** 10.1186/s12970-019-0321-8

**Published:** 2019-11-10

**Authors:** Santiago Sanz-Quinto, Manuel Moya-Ramón, Gabriel Brizuela, Ian Rice, Tomás Urbán, Raúl López-Grueso

**Affiliations:** 1 Department of Sport Sciences, Miguel Hernandez University, Elche, Av. de la Universidad s/n, 03202 Elche, Alicante Spain; 2 Department of Sport Sciences, Miguel Hernandez University, Elche, Institute for Health and Biomedical Reaearch (ISABIAL-FISABIO), 03010 Alicante, Spain; 3 Department of Physical Education and Sports, University of Valencia, Gasco Oliag, 3, 46010 Valencia, Spain; 4 Department of Kinesiology and Community Health College of Applied Health Sciences, University of Illinois at Urbana-Champaign 2003 Huff Hall, M/C 586, 1206 S. Fourth St, Champaign, IL 61820 USA

**Keywords:** Hypoxia, Nutritional intervention, Paralympic, Energy intake, Body mass

## Abstract

**Background:**

Altitude training is a common practice among middle-distance and marathon runners. During acclimatization, sympathetic drive may increase resting metabolic rate (RMR), therefore implementation of targeted nutritional interventions based on training demands and environmental conditions becomes paramount. This single case study represents the first nutritional intervention performed under hypobaric hypoxic conditions (3900 m) in Paralympic sport. These results may elucidate the unique nutritional requirements of upper body endurance athletes training at altitude.

**Case presentation:**

This case study examined the effects of a nutritional intervention on the body mass of a 36-year-old professional wheelchair athlete (silver medalist at the Paralympic Games and 106 victories in assorted road events) during a five-week altitude training camp, divided into pre-altitude at sea level (B_N_), acclimatization to altitude (Puno, 3860 m) (B_H_), specific training (W_1,2,3,4_) and return to sea level (Post) phases. Energy intake (kcal) and body mass (kg) were recorded daily. Results demonstrated significant decrease in body mass between B_N_ and B_H_ (52.6 ± 0.4 vs 50.7 ± 0.5 kg, *P* < 0.001) which returned to pre-altitude values, upon returning to sea level at Post (52.1 ± 0.5 kg). A greater daily intake was observed during B_H_ (2899 ± 670 kcal) and W_1,2,3_ (3037 ± 490; 3116 ± 170; 3101 ± 385 kcal) compared to B_N_ (2397 ± 242 kcal, *P* < 0.01) and Post (2411 ± 137 kcal, *P* < 0.01). No differences were reported between W_4_ (2786 ± 375 kcal), B_N_ and Post. The amount of carbohydrates ingested (g · kg^− 1^) was greater in W_1,2,3,_ (9.6 ± 2.1; 9.9 ± 1.2; 9.6 ± 1.2) than in B_N_ (7.1 ± 1.2) and Post (6.3 ± 0.8, *P* < 0.001). Effect sizes (Cohen’s *d*) for all variables relative to B_N_ (all time points) exceed a large effect (d > 0.80).

**Conclusions:**

These results suggest an elite wheelchair marathoner training at 3860 m required increased nutrient requirements as well as the systematic control needed to re-adapt a nutritional program. Moreover, our findings highlight training and nutritional prescription optimization of elite wheelchair athletes, under challenging environmental conditions.

## Background

In recent years, there has been emerging interest in the optimization of nutritional strategies to help athletes reach their fitness goals during hypoxic training conditions [1]. However, nutritional guidelines for athletes training at 4000 m altitude remain unclear as most nutritional and exercise metabolism studies have been completed at lower altitudes [1, 2], and the data reflects athletes participating in activities less than marathon distances [3–9]. For example, in distance running only one study has examined well-trained runners at an altitude of 4000 m [10] and, recently a case study reported physiological data on an elite wheelchair marathoner training at 3900 m altitude [11]. Loss of body fat and fat free mass have been reported during high altitude sojourns in people eating ad libitum [12–15], suggesting that strict altitude imposed dietary controls can attenuate daily energy deficits and partially mitigate weight loss [16]. Loss of fat free mass at high altitude increases the risk of illness and injury in extreme environments [5, 17–19]. During acclimatization there is a reduction of intra and extracellular water combined with a decrease in plasma volume [6, 20], which can result in body mass loss up to 2 kg [14]. Furthermore, during acute phase exposure, total exogenous glucose oxidation appears to be lower than at sea level, and after 21 days of initial exposure at 4300 m not reaching sea level, suggesting oxidation rates under hypoxic conditions do not cover the energy demands of athletes at altitude [9]. Alternatively, other studies suggest individuals have an increased dependence on glucose as a fuel source at high altitude, especially during exercise [3, 7, 8].

Increased resting metabolic rate (RMR) has also been observed at altitude, which could be due to increased sympathetic drive and subsequent rise in adrenaline levels [21]. Recent research found that RMR in elite middle-distance runners increased by ≈ 19% at a moderate altitude (2100 m) compared to sea-level conditions [2] and 10% at high altitude (3800 m) [22]. In contrast, a small decrease in RMR was reported in a group of Olympic rowers training at 1800 m [23]. Moreover, RMR is more pronounced over the first 2–3 days after arrival [16, 24]. However, elevated RMR (≥ 17%) can persist for up to 21 d after initial high altitude exposure [17]. Ultimately, energy expenditure which is elevated at altitude may be equivalent to high intensity exercise conducted at sea level [25].

Due to the aforementioned factors, one of the primary nutritional goals for managing a successful altitude training camp involves matching the energy intake to the daily expenditure in order to minimize body mass loss [26]. In fact, it was reported that, a total of 7.6 g · kg^− 1^ body mass of carbohydrates (CHO) per day did not cover the energetic demands of cyclists living and training at 4300 m [4]. Importantly, up to 70% of the chronic altitude exposure-related weight loss is said to be due to reductions in muscle mass itself [27]. To consider, D’Hulst & Deldique [28] recently suggested that based on the hypoxic dose theory [29], an exposure of 5000 km · h^− 1^ is the cutoff point above which muscle loss starts to occur. However, at altitude the stimulation of protein synthesis after exercise might be blunted by hypoxia, as it was shown that increase in muscle protein synthesis following walking at 4559 m [30] was much lower than a comparable study with exercise performed at sea level [31]. Interestingly, in a separate study, body mass was maintained in ski mountaineers following an isocaloric diet of 4000 kcal · d^− 1^, supplemented with 1.5 g or 2.5 g · kg body mass casein protein per day during seven days at 2500–3800 m [32]. Moreover Bigard and colleagues examined the effects of branch chain amino acids (BCAA) (7.8 g leucine, 3.4 g isoleucine, 11.2 g valine; 1.44 g protein · kg · d) compared to carbohydrate supplementation on body composition following six days of ski mountaineering at 2500–3800 m. Body composition and muscular performance were unaffected by BCAA. However, significant weight loss only occurred in the carbohydrate-supplemented group (− 1.55 vs. -0.8 kg) [32].

The purpose of this study was to examine the effects of a nutritional intervention on the body mass of an elite wheelchair marathoner during a five-week training camp performed between sea level and 3900 m altitude. The intervention was designed to anticipate increases in RMR due to the combined effects of both environmentally induced hypoxia and the demands of marathon training.

## Case presentation

The study athlete was a 36-year-old, elite wheelchair marathoner, functional class T52 (upper limb involvement category). Some of his accolades include winning a silver medal at the Paralympic Games and 106 victories in assorted road events, including a win at the 2016 Boston Marathon, ten weeks after returning to sea level from Los Andes (Peruvian Altiplano). Our participant’s height = 1.76 m; body mass = 52.6 ± 0.4 kg; power output at second ventilatory threshold = 62 W; training 8000 km per year; former world record holder in the T52 division in 800 m (1 min:56 s); 1500 m (3 min:36 s); world record holder in 5000 m (12 min:37 s); half marathon (50 min:28 s) and fourth best ever time in marathon (1 h:42 min:05 s). Additionally, he has more than ten years of altitude training experience, with training camps performed in Boulder, CO (1655 m), Navacerrada, Spain (1858 m), Flagstaff, AZ (2106 m), Sierra Nevada, Spain (2320 m), Keystone, CO (2796 m) and Breckenridge, CO (2926 m), performing both altitude models: Live-High-Train-High (LHTH) and Live-High-Train-Low (LHTL) and has been exposed to more than 8000 h of normobaric-hypoxia. For the last five seasons prior to the current study, the athletes trained at moderate altitudes (1655 up to 2926 m) for: 78, 82, 101, 79 and 62 days.

The athlete requested advice for the development of an individualized nutritional program based on training loads to prepare for his upcoming season. Therefore, after consultation with laboratory members a nutrition program was designed, according to his training load (Table 1).

**Table 1:** Main meals designed for each type of session under altitude conditions

Session	Breakfast	Lunch	Dinner	Energy Intake (kcal)	Carbohydrate Protein Fat (g)
A	62 g cereals, 204 g soy milk, 26 g white bread, 18 g jam, 3 g black tea in ~ 200 ml water, 12 g sugar	180 g (dry) spaghetti, ~ 150 g alpaca, 8 g olive oil	180 g (dry) steamed rice, 180 g emperor fish, 10 g olive oil	2393	383 111 49
B	62 g cereals, 204 g soy milk, 3 g black tea in ~ 200 ml water, 12 g sugar	180 g (dry) spaghetti, ~ 130 g alpaca, 8 g olive oil	180 g (dry) steamed rice, 180 g emperor fish, 10 g olive oil, 8 g parmesan cheese	2357	353 116 52
C	80 g cereals, 200 g soy milk, 3 g black tea in ~ 200 ml water, 12 g sugar	180 g (dry) spaghetti, 140 g beef, 10 g olive oil, 14 g parmesan cheese	180 g (dry) spaghetti, 125 g tuna, 12 g olive oil, 10 g parmesan cheese, 180 g fat free yoghurt	2424	365 119 42
D	80 g cereals, 200 g soy milk, 130 g coffee, 25 g fat free milk, 14 g sugar	180 g (dry) spaghetti, ~ 150 g alpaca, 8 g olive oil, 8 g parmesan cheese	140 g (dry) rice, 120 g omelette, 170 g tuna canned	2639	348 135 75
E	80 g cereals, 204 g soy milk, 3 g black tea in ~ 200 ml water, 12 g sugar	160 g (dry) rice, ~ 160 g chicken breast, 14 g olive oil, 1 kiwi	180 g (dry) spaghetti, 135 g tuna, 20 g parmesan cheese, 6 g olive oil	2351	374 114 36
F	80 g cereals, 204 g soy milk, 3 g black tea in ~ 200 ml water, 10 g sugar	180 g (dry) spaghetti, ~ 160 g chicken breast, 14 g olive oil, 8 g parmesan cheese, 180 g fat free yoghurt	170 g alpaca, 275 g sweet potato	2091	282 143 43
G	50 g cereals, 200 g soy milk, 17 g coffee, 25 g fat free milk, 12 g sugar	180 g fat free yoghurt 140 g (dry) rice, 120 g omelette, 12 g olive oil	500 g Margherita (pizza)	2618	343 113 89

Session A: 20 × 400 m ~ VT2; Session B: 2 h ~ VT1; Session C: 6 × 2000 m ~ VT2; Session D: 20 km < VT1 in the morning + 16 km < VT1 in the afternoon; Session E: 16 km < VT1 in the morning + Gym session in the afternoon; Session F: 20 km < VT1 in the morning; Session G: Day off

The research participant provided written consent prior to participation in the current study and read the manuscript before submission. Research was approved by the Ethics Research Committee of the University Miguel Hernandez.

### Training protocol

Both pre-altitude (B_N_), at 16 m and acclimatization (B_H_) at 3900 m incorporated identical training loads (128 km of mileage each). However, the first two days of B_H_ incorporated no training to minimize the effects of jet-lag, and acute mountain symptoms (AMS), like headache [33]. Two daily training sessions were performed from Wednesday to Friday under the first ventilatory threshold (<VT1). The morning session involved 20 km of distance training and the afternoon session 16 km. A 20 km workout was performed on Saturday <VT1. Sunday was a rest day. Specific training weeks “W_1_, W_2_, W_3_ & W_4_” were based on a day-to-day basis periodization, according to level of heart rate variability (HRV) [34]. When the HRV reached a reference value (RV), the subject completed a specific session in the morning, followed by an evening off. If the RV was not reached, two workouts <VT1 were performed: 20 km in the morning and 16 km in the afternoon. On three days the training was fixed; On Mondays and Thursdays the AM sessions were 16 km < VT1, while the PM sessions involved resistance training and Sundays were off. The specific sessions were known as: A (20 × 400 m at ~ second ventilatory threshold (VT2) in a plateau at 4090 m altitude; recovery reps: 75 s); B (30 km ~ VT1) and C (6 × 2000 m ~ VT2 in a plateau at 4090 m altitude; recovery reps: 120 s).

As a way to induce muscle hypertrophy, resistance sessions were performed at 80% of 1 RM [35] with 4 sets of 8 reps with 150 s recovery, aimed at avoiding loss of muscle mass induced by chronic hypoxia. RM test was not performed under altitude conditions due to high risk of injury, so it was done four days before flying to Peru. More details on the experimental design have been reported previously [11].

### Daily recording

**Fig. 1:**
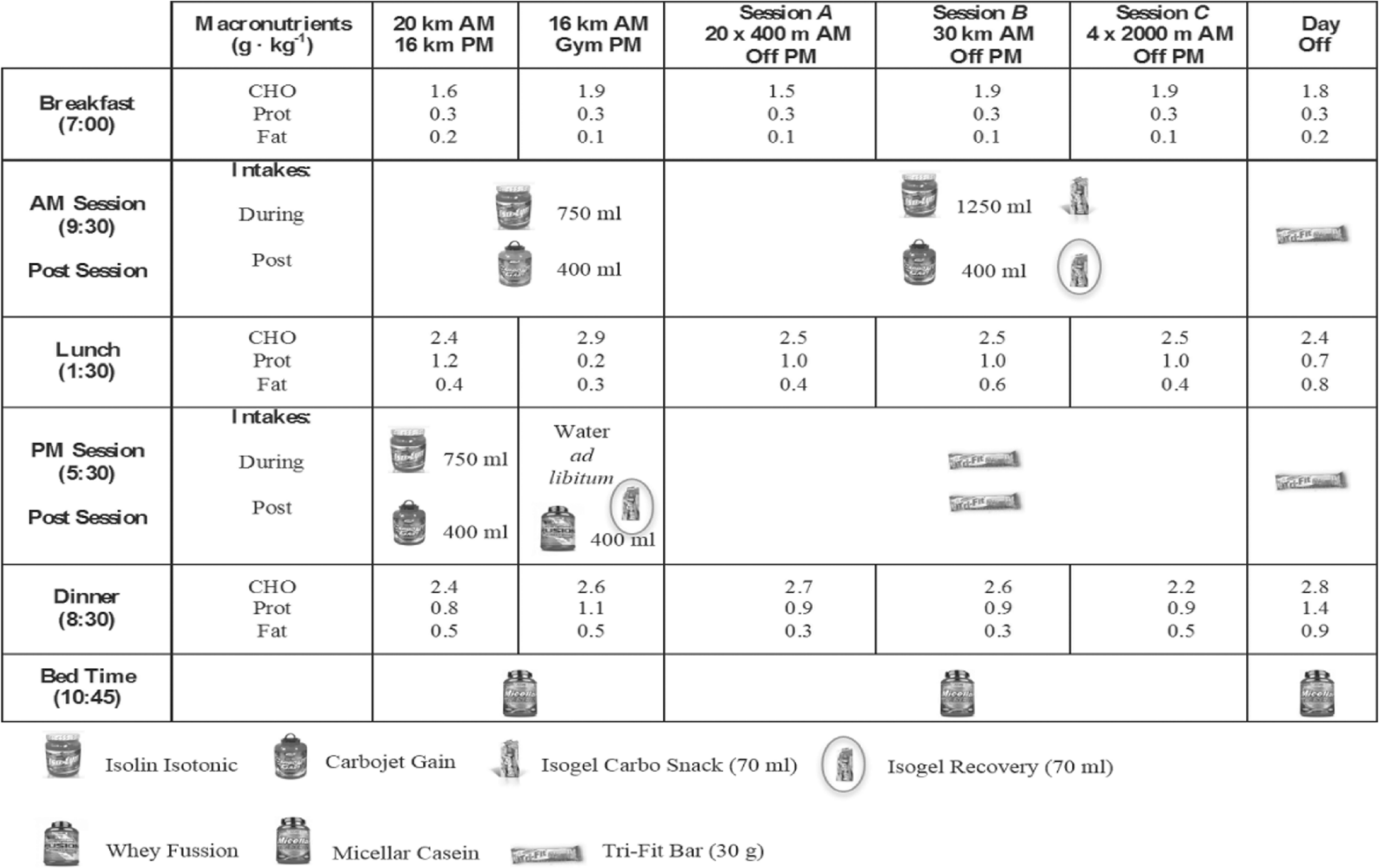
Timing of Daily Food and Fluid Intake during Altitude, based on different training routines

**Fig. 2:**
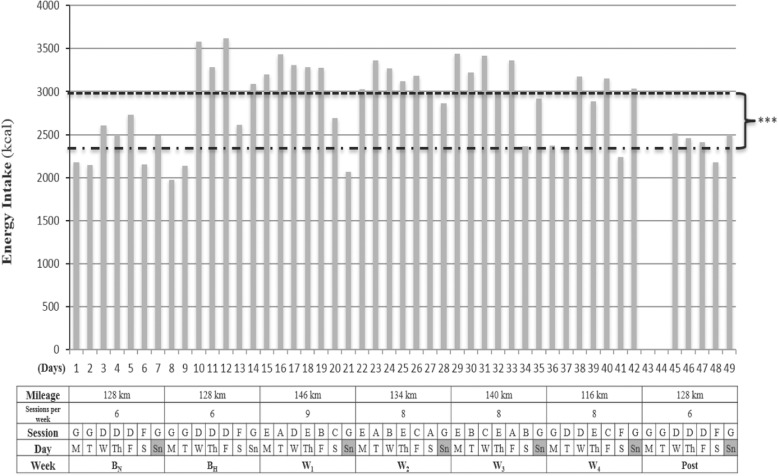
Training program and energy intake during B_N_, B_H_, W_1,2,3,4_ and Post. B_N_, baseline in normoxia; B_H_, baseline in hypoxia; W_1,2,3,4_, specific training weeks in hypoxia; Post, returning sea level week. Session A: performed on a plateau at 4090 m; 8 km + technique drills + 5 × 80 m accelerations + 20 × 400 m ~ VT2 + 2 km. Recovery reps 75 s. Session B: 2 h ~ VT1. Session C: performed on a plateau at 4090 m; 8 km + technique drills + 5 × 80 m accelerations + 6 × 2000 m ~ VT2 + 2 km. Recovery reps 120 s. Session D: 20 km < VT1 in the morning + 16 km < VT1 in the afternoon. Session E: 16 km < VT1 in the morning + gym session in the afternoon (4 sets × 8 reps recovery sets 150 s at 80% RM). Exercises for resistance session: press bench, close grip, dumbbell press, seated military press and seated cable row). Session F: 20 km < VT1 in the morning + resting afternoon. Session G: Day off. Dash line: Represent mean energy intake (2423 kcal) in normoxic conditions at sea level. Round dot line: Represent mean energy intake (3017 kcal) in hypoxic conditions at 3900 m altitude. Differences from mean energy intake under hypoxic conditions: *** *P* < 0.001

Throughout the experiment, basal body mass was recorded in fasting conditions, naked, after waking up, with a digital scale (Tanita BC-601®, TANITA Corporation, Tokyo, Japan). Utilizing a food recording system previously reported [1], a nutritional diary was maintained by the subject to record daily intake, which included main meals (breakfast, lunch and dinner), two small snacks and all training activities that occurred post-intake (Figs. 1 and 2).

Total energy (kcal), carbohydrates, proteins and fats (g · kg^− 1^ body mass) were estimated according to nutritional composition database supported by the Spanish Ministry of Science and Innovation [36].

### Nutritional program

The athlete was instructed by a nutritionist to prepare all meals which included weighing both ingredients prior to cooking and left overs prior to disposal. On days when the athlete ate at restaurants, which occurred on four occasions, he was instructed to send pictures of these meals to the research team [37]. A personal chef was contacted to buy and cook all foods/ingredients for the athlete on a daily basis according to athlete instructions while the weighing and cooking process occurred under the athlete’s supervision. Additionally, the athlete was instructed to prepare all training drinks and post-training recovery solutions. To prevent contamination, the athlete did not eat raw foods or unpeeled fruits or vegetables and no water from the tap was consumed [38]. At sea level the athlete cooked all meals at home.

Daily energy intake was increased ~ 20% from pre-altitude (B_N_), to arrival at altitude (B_H_) to avoid body mass loss from increased RMR which is common while living and training at higher altitudes [2, 22]. Moreover, main meals were designed according to the type of training session performed (Fig. 2), as we have recently reported that during specific training weeks (W_1,2,3,4_) number of A,B,C, sessions differed between specific training weeks, according to a training program based in HRV [11], which explains why at W_2_ the greatest amount of CHO was ingested (9.9 ± 1.2 g · kg^− 1^ body mass), and why during B_H_ and W_4_ the total amount of CHO tended to be lower than W_1,2,3_ ( Table 2). Moreover, main meals were accompanied by two rich-carbohydrate snacks, based on reports that the inclusion of several rich carbohydrate snacks, more optimally covers increased energy requirements than three standalone main meals [38]. Furthermore, regarding proteins, a minimum intake of 2.4 g · kg^− 1^ body mass was targeted in the current nutritional design to avoid loss of lean mass [39]. To avoid gastrointestinal issues (GI) and fullness [40], a low protein/fat intake was provided for breakfast and PM sessions, however the percentage of lipids at lunch was lower than dinner. Protein intake at lunch and dinner were ≈ 1 g · kg^− 1^, given that specific and, more demanding sessions (A,B,C) were performed in the morning, and muscle tissue repair is a main meal target. The ingestion of lipids was set at a minimum of 1 g · kg^− 1^ body mass throughout the sea level and altitude camps, as fat cells increase their sensitivity to hormonal stimulation after training, resulting in a greater mobilization of fatty acids [41]. Moreover, an Iso-Lyn Isotonic (AMIX) sports drink was used for workouts < VT1 shorter than 65 min (20 and 16 km). The athlete was instructed to drink a solution with 750 ml of water and 56.4 g of CHO, while a solution of 1250 ml with 80 g of CHO was recommended for specific sessions. The CHO rate was 0.5 to 1 g · kg^− 1^ body mass per hour [42]. Despite these recommendations, the athlete and team elected to preserve his natural drinking habits that involved consuming drinks every 10 min. This decision was made because fluid consumption for a wheelchair racer can be precarious during propulsion, as they must come out of their natural prone/kneeling body position to drink. This action can force loss of vision, which increases the risk of collision or crashing. Because our participant never experienced GI in his career with the use of carb gels [43], he drank a 42 g CHO (Glucose + Fructose) Iso-Gel carbo snack (AMIX) during specific sessions workouts [44]. Gels were consumed in the A session after fourteen 400 m rep, in the B session 90 min after starting, and in the C session after four 2000 m rep. Both types of carbs used in the solution and gels were multiple transportable carbohydrates, as directed by Jeukendrup [45].

During gym sessions water was consumed ad libitum and immediately after gym sessions the athlete co-ingested a rich leucine whey protein (23.6 g) (Whey Fussion, AMIX) dissolved in 400 ml of water and a carbohydrate gel (Iso-Gel Recovery, AMIX) (37.6 g maltodextrin + fructose + Vitargo®) as directed for speeding up to 25% glycogen synthesis [46]. For refueling purposes carbohydrate guidelines [42], suggest aiming for post-exercise rapid recovery of muscle glycogen deposits, with 1 g · kg^− 1^ body mass of CHO, repeated every 2–3 h. After specific sessions, a carbohydrate shake was taken with a carbohydrate gel, providing 1.4 g · kg^− 1^ body mass. In the hour immediately after 16 km and 20 km < VT1, the subject drank a carbohydrate solution (Carbojet Gain, AMIX) (34 g CHO, 7.5 g prot, 1.8 g fat) dissolved in 400 ml of water, and after specific sessions he ingested a combination of the same drink plus Iso-Gel Recovery. To consider, 2.4 g · kg^− 1^ body mass, CHO were consumed (Fig. 1) at lunch which occurred approximately two hours post-exercise meal, in order to achieve 3.1 g · kg^− 1^ body mass of CHO 3 h post-exercise for our athlete vs. 3 g · kg^− 1^ body mass as suggested by Burke and colleagues [42].

On specific session days, rest was provided in the evenings along with a snack at 5:30 PM, to meet increased energy requirements [38]. This snack included two 30 g cereal bars (Tri-Fit Bar, AMIX) (34.9 g CHO, 3.9 g prot, and 10.1 g fat).

In a manner to avoid loss of body mass [32] and enhance muscle protein synthesis [47] the athlete consumed 2.5 g leucine, 1.5 g isoleucine, and 1.5 g valine) immediately after each session (BCAA Elite Rate, AMIX). Before bedtime, 30 g of casein protein (Micellar Casein, AMIX) (1.7 g CHO, 24 g prot, 0.6 g fat) was ingested as suggested by Snijders and colleagues [48].

Finally, the athlete maintained iron levels through a daily intake of 105 mg of ferrous sulphate (Ferogradumet®, Ross, Abbott Científica), as ferrous sulphate intake has been related to the production of Hemoglobin and red cells [49, 50]. To comply with World Anti-Doping Agency (WADA) regulations, none of the aforementioned supplements contain prohibited substance.

For a description of the macronutrients intake during main meals in each session see Fig. 1.

### Statistical analysis

All data are presented as mean ± SD. A repeated-measures ANOVA was carried out for all the variables including the factor TIME with levels B_N_, B_H_, W_1_, W_2_, W_3_, W_4_ and Post. A post hoc least significance difference (LSD) multiple-range test was performed to determine differences between the factor levels. Effect size (d) associated with change in body mass was calculated using Cohen’s d (difference in mean scores over time divided by pooled SD) with its 95% confidence limits (CL) [51] and were interpreted as trivial (≤ 0.19), small (0.20–0.49), medium (0.50–0.79), and large (≥ 0.80) [52]. An alpha level of 0.05 was stated for statistical significance. Statistical analyses were performed using the SPSS version 22.0 (SPSS, Inc., Chicago, IL, USA) software.

## Results

Our nutritional intervention results can be found in Table 2.

**Table 2:** Body mass and nutritional parameters during sea level and altitude

Phase	Body Mass (kg)	Daily Intake (kcal)	CHO (g · kg^− 1^)	Prot (g · kg^− 1^)	Fat (g · kg^− 1^)
B_N_	52.6 ± 0.4 (52.25; 53.04)	2397 ± 242 (2173; 2621)	7.1 ± 1.2^ijk^ (5.97; 8.19)	1.9 ± 0.2^hi^ (1.74; 2.11)	1.0 ± 0.2 (0.82; 1.14)
B_H_	50.7 ± 0.5^g^ (50.23; 51.17)	2899 ± 670^a^ (2280; 3518)	8.1 ± 2.2^j^ (6.04; 10.11)	2.9 ± 0.5 (2.46; 3.38)	1.4 ± 0.5 (0.92; 1.89)
W_1_	50.6 ± 0.2^g^ (50.39; 50.78)	3037 ± 490^a^ (2584; 3490)	9.6 ± 2.1 (7.68; 11.55)	2.7 ± 0.5 (2.18; 3.17)	1.2 ± 0.4 (0.81; 1.49)
W_2_	50.8 ± 0.4^g^ (50.45; 51.09)	3116 ± 170^a^ (2959; 3273)	9.9 ± 1.2 (8.79; 11.09)	2.6 ± 0.4^g^ (2.17; 2.99)	1.1 ± 0.5 (0.65; 1.60)
W_3_	50.9 ± 0.3^g^ (50.68; 51.15)	3101 ± 385^a^ (2744; 3457)	9.6 ± 1.2 (8.53; 10.73)	2.7 ± 0.5^g^ (2.25; 3.22)	1.2 ± 0.5 (0.75; 1.64)
W_4_	51.2 ± 0.3^ghi^ (50.93; 51.47)	2786 ± 375 (2439; 3133)	8.6 ± 1.3 (7.39; 9.73)	2.5 ± 0.3^g^ (2.21; 2.77)	1.1 ± 0.5 (0.57; 1.56)
Post	52.1 ± 0.5^ghijkl^ (51.54; 52.66)	2411 ± 137^bcde^ (2241; 2580)	6.3 ± 0.8^ijkl^ (5.41; 7.27)	1.9 ± 0.3^hijkl^ (1.55; 2.31)	1.3 ± 0.3 (0.94; 1.69)

B_N_, Baseline in normoxia at 16 m altitude; B_H_, Baseline in hypoxia at 3860 m altitude; W_1,_ First week of specific training; W_2,_ Second week of specific training; W_3,_ Third week of specific training; W_4,_ Fourth week of specific training; Post, values after altitude training camp at 16 m altitude; Body Mass: wake up body mass, kg; Daily intake, is the amount of daily kilocalories; CHO, is the amount of daily carbohydrates related to the AM body mass; Prot, is the amount of daily proteins related to the AM body mass; Fat, is the amount of daily fats related to the AM body mass; Mean ± SD (95% CL)

Differences from B_N_: ^a^ *P* < 0.01; ^g^ *P* < 0.001;

Differences from B_H:_^b^ *P* < 0.01; ^h^ *P* < 0.001;

Differences from W_1:_^c^ *P* < 0.01; ^i^ *P* < 0.001;

Differences from W_2:_^d^ *P* < 0.01; ^j^ *P* < 0.001;

Differences from W_3:_^e^ *P* < 0.01; ^k^ *P* < 0.001;

Differences from W_4:_^f^ *P* < 0.01; ^l^ *P* < 0.001

### Body mass

A significant decrease in body mass was observed from B_N_ to B_H_ [*P* < 0.001; d = 4.16, 95% CL (2.02; 5.71)] but returned to near baseline levels during Post. There were no significant effect for time during the W_1,2,3_ period, however we observed a significant increase in body mass from W_1_ to W_4_ [*P* < 0.001; d = 2.35, 95% CL (0.86; 3.51)].

### Energy intake

Results show a greater amount of kcal in B_H_ [*P* < 0.01; d = 0.96, 95% CL (− 0.25; 2.04)] and W_1_ [*P* < .01; d = 1.61, 95% CL (0.27; 2.73)], W_2_ (*P* < 0.01; d = 3.49, 95% CL (1.59; 4.91)], W_3_ [*P* < 0.01; d = 2.15, 95% CL (− 0.66; 3.33)] than in B_N_. Same differences were observed within B_H_ [*P* < 0.01; d = 0.97, 95% CL (− 0.24; 2.05)], W_1_ [*P* < 0.01; d = 1.68, 95% CL (0.31; 2.80)], W_2_ [*P* < 0.01; d = 4.52, 95% CL (2.26; 6.16)], W_3_ [*P* < 0.01; d = 2.31, 95% CL (0.78; 3.51)] and Post. No differences were reported between W_4_, B_N_ and Post.

### Carbohydrates

The amount of CHO ingested (g · kg^− 1^ body mass) was greater in W_1_ [*P* < 0.001; d = 1.43, 95% CL (0.12; 2.53)], W_2_ [*P* < 0.001; d = 2.33, 95% CL (0.80; 3.54)], W_3_ [*P* < 0.001; d = 2.08, 95% CL (0.62; 3.26)] than in B_N_. Differences were observed within W_1_ [v0.01; d = 2.01, 95% CL (0.56; 3.17)], W_2_ [*P* < 0.01; d = 3.47, 95% CL (1.58; 4.88)], W_3_ [*P* < 0.01; d = 3.18, 95% CL (1.38; 4.53)] and Post.

### Proteins

Protein intake (g · kg^− 1^ body mass) was greater in B_H_ (*P* < 0.001; d = 2.54, 95% CL (0.95; 3.79)] and W_1_ (*P* < 0.001; d = 2.03, 95% CL (0.58; 3.20)], W_2_ (*P* < 0.001; d = 2.16, 95% CL (0.67; 3.34)], W_3_ (*P* < 0.001; d = 2.03, 95% CL (0.58; 3.20)], W_4_ (*P* < 0.001; d = 2.31, 95% CL (0.78; 3.52)] than in B_N_. Same differences were found within B_H_ (*P* < 0.01; d = 2.38, 95% CL (0.83; 3.59)], W_1_ (*P* < 0.01; d = 1.90, 95% CL (0.48; 3.05)], W_2_ (*P* < 0.01; d = 1.96, 95% CL (0.52; 3.11)], W_3_ (*P* < 0.01; d = 1.90, 95% CL (0.48; 3.05)], W_4_ (*P* < 0.01; d = 2.00, 95% CL (0.56; 3.16)] and Post.

### Lipids

No differences were found in lipids intake (g · kg^− 1^ body mass) within any period.

## Discussion

The aim of this case study was to assess the effectiveness of an evidence based individualized nutrition program applied to an elite wheelchair marathoner during a five-week altitude training camp, carried out in the Peruvian Altiplano (Puno, Peru) at 3900 m. The program was designed based on existing literature for its ability to sustain the athlete’s body mass and meet the energetic demands of intense training, while promoting substrate availability, nutrient recovery, and muscle tissue repair. Interestingly, the designed nutritional intervention helped to: 1) maintain the athlete’s body mass throughout the altitude camp, 2) minimize performance deficits during intense training at altitude compared to sea level (~ 20 to ~ 24% in 1609 m and 3218 m reps respectively) [10], as evidence by recently reported data demonstrating a ~ 3% reduction in reps (2000 m) [11], 3) facilitate intra-sessions recovery through faster glycogen restoration, helping the athlete to perform during physiological demanding sessions (~ VT2) when completed consecutively, or until two sessions of ~ 2 h at ~ VT1 at W_2_ [11], and 4) maintain quality training sessions at altitude as evidence by: a) improved power output, 11-d post-altitude compared to 4-d pre-altitude (44 W vs 50 W), b) time reductions during 3000 m races 12-d post-altitude compared to 3-d pre-altitude (472 s vs 456 s) [11].

At 4300 m there can be an increase in respiratory water loss, due to greater ventilation and an increase in urinary water loss that can increase up to 500 ml per day [17]. This could explain the nearly 2 kg weight loss observed from baseline (B_N_) to acclimatization phase (B_H_) and the return to pre-altitude levels in post (Table 2). It should be noted that there was an increment of energy intake of ≈ 500 kcal in hypoxic conditions compared to normoxic conditions (*P* = 0.001) and same training was done in B_N_ and B_H_ (Fig. 2). Of note, all effect sizes associated with statistically significant changes in body mass far exceeded Cohen’s convention for a large effect.

Increased RMR has been reported in athletes who live and train at altitude [2]. For this reason, to maintain body mass in the current study, there was a significant increase in the amount of carbohydrates per kilogram of body mass and proteins per kilogram of body mass provided at altitude compared to sea level. We suspect that the slight increase in body mass observed in W_4_ was induced by the different number of specific sessions performed from W_1_ to W_4_; 2 in W_1_, 3 in W_2_, 2 in W_3_ and 1 in W_4_ [11]. To increase energy supply, as a result of a greater energy demands and to avoid GI, six meals (breakfast, post-training AM, lunch, snack or post-training PM, dinner and bedtime) were projected in an elapsed time within three hours each one (Fig. 1), as it has been recommended to include several rich carbohydrate snacks, rather than three main meals [38]. We did not find differences in energy intake between acclimatization (B_H_) and specific training weeks (W_1_ to W_4_) however this could be due to the fact that when the athlete performed a specific session in the morning, a rest afternoon was followed, in spite of two sessions performed daily during acclimatization with 36 km volume (Fig. 2). Furthermore, we did not consider a slightly lower exogenous glucose oxidation rate during acclimatization and chronic altitude [53], as it has been reported that such observations should be contrasted with fully fed individuals, although evidence exists to the contrary [3, 8]. Three hours before training sessions, a rich CHO meal was consumed, as it has been shown to increase glycogen availability [42]. We recommended that the athlete change from cereals to a lower fiber food like white bread to avoid GI distress however because of disability imposed manual dexterity deficits which prevent cutting bread slices and spreading fruit jam, he decided to use cereals. The research team also had to consider that the athlete ate breakfast by seven in the morning, which was nearly two and a half hours before training sessions. However, the athlete commonly practiced training in a fasted state like this during training sessions at home, to minimize GI. Despite the athlete’s comfortability with this practice, it was discarded in Puno because temperatures were extremely cold by 7 am (~ 0 °C) and he trained barefoot.

To avoid a loss of muscle mass, high-protein foods were spread out across all meals (Fig. 1), while whey and casein protein training products were consumed to ensure minimum requirements of 2.4 g · kg^− 1^ body mass were achieved [39]. However, we have to consider that the hypoxic dose [29] of this training camp was 3300 km · h^− 1^, not reaching the cut off point, where muscle loss begins [28]. Due to personal preferences, protein delivery by meat was introduced at lunch, while fish was eaten at dinner. No eggs were eaten while training however the athlete ate an omelet for lunch during rest days (Table 1).

### Limitations

Main limitations of this study are evident in the absence of outcomes like upper body skinfolds, and upper arm circumference measurements, which could help us to know if body fat percentage and loss of muscle mass occurred in our athlete which was reported previously in subjects eating ad libitum under hypoxic conditions [12–15]. Moreover, RMR was not assessed, as recently reported [23] in Olympic rowers training at 1800 m who did not show an increase in RMR. However, our athlete was exposed to more intense hypoxic conditions, so sympathoexcitation may have occurred [54] leading to elevated adrenaline levels and subsequent greater energetic demands. Another limitation was evident in the use of a self-reported intake diary conducted without supervision from a nutritionist, however the athlete was provide instructions for meal preparation as described previously. Importantly, similar self reported nutritional tools have been validated for estimating energy and nutrient intake [37]. Also, the use of pictures on four occasions to record restaurant meal consumption must be considered as a limitation. However, this methodology has been supported by exercise nutritionists as a useful strategy, particularly when research teams are not present [1]. Finally, the absence of muscular biopsies did not allow us to measure glycogen and protein muscle content.

## Conclusions

The aim of the daily meal distributions (Fig. 1) was to cover the energetic demands of training sessions and to ensure substrate availability, nutrients recovery, and muscle tissue repair according to literature recommendations.

This paper can help us to better understand the unique nutritional requirements of upper body endurance athletes during altitude training conditions where nutritional strategies may differ from able-bodied athletes. Importantly, to confirm and expand on the current findings specific to the aforementioned differences between able bodied and upper limb athletes, more research is needed on both populations. However, analogous studies are scarce in able bodied athletes and nonexistent in upper limb athletes. For example, only one study, published in 1967 examined well-trained athletes at 4000 m [10], while others have investigated nutritional interventions or exercise metabolism at moderate altitudes only (2150 m) [1, 2]. To date, the only other studies conducted at altitudes similar to ours involved either dissimilar sports disciplines [4], lacked a nutritional component [10], or utilized none elite athletes [53]. Ultimately, this study represents the first nutritional intervention conducted on an elite wheelchair marathoner under altitude conditions. Since no specific nutritional interventions have been performed on able-bodied marathon runners or wheelchair athletes at 4000 m altitude, all nutritional guidelines were reflective of the literature pertaining to able-bodied athletes training at lower altitudes.

Ultimately, our nutritional intervention targeted body mass maintenance to sufficiently anticipate increases in RMR due to the combined effects of environmentally induced hypoxia and the demands of marathon training. Moreover, the intervention helped minimize performance perturbations, facilitated overall recovery, and enhanced athlete performance post-altitude. Future related studies should be designed based on considerations from the current study, however with more specificity therefore utilizing deeper assessment tools like biological samples. For example biopsies could be applied to determine the protein and glycogen synthesis-breakdown cycle of athletes during periods of intense training.

## Data Availability

Please contact authors for data requests.

## Abbreviations

AMS: Acute mountain symptoms
BCAA: Branch chain amino acids
B_H_: Altitude acclimatization
B_N_: Pre-altitude
CHO: Carbohydrates
CL: Confidence limits
GI: Gastrointestinal issues
HRV: Heart rate variability
LHTH: Live-High-Train-High
LHTL: Live-High-Train-Low
RMR: Resting metabolic rate
RV: Reference value
SD: Standard Deviation
VT1: First ventilatory threshold
VT2: Second ventilatory threshold
W_1_,W_2_, W_3_, W_4_: Specific training weeks at altitude
